# Modeling EHR with the openEHR approach: an exploratory study in China

**DOI:** 10.1186/s12911-018-0650-6

**Published:** 2018-08-29

**Authors:** Lingtong Min, Qi Tian, Xudong Lu, Huilong Duan

**Affiliations:** College of Biomedical Engineering & Instrument Science, Zhejiang University, Zheda Road, Hangzhou, 310027 China

**Keywords:** OpenEHR approach, Electronic health record, Archetypes, Modeling EHR

## Abstract

**Background:**

The openEHR approach can improve the interoperability of electronic health record (EHR) through two-level modeling. Developing archetypes for the complete EHR dataset is essential for implementing a large-scale interoperable EHR system with the openEHR approach. Although the openEHR approach has been applied in different domains, the feasibility of archetyping a complete EHR dataset in a hospital has not been reported in academic literature, especially in a country where using openEHR is still in its infancy stage, like China. This paper presents a case study of modeling an EHR in China aiming to investigate the feasibility and challenges of archetyping a complete EHR dataset with the openEHR approach.

**Methods:**

We proposed an archetype modeling method including an iterative process of collecting requirements, normalizing data elements, organizing concepts, searching corresponding archetypes, editing archetypes and reviewing archetypes. Two representative EHR systems from Chinese vendors and the existing Chinese EHR standards have been used as resources to identify the requirements of EHR in China, and a case study of modeling EHR in China has been conducted. Based on the models developed in this case study, we have implemented a clinical data repository (CDR) to verify the feasibility of modeling EHR with archetypes.

**Results:**

Sixty four archetypes were developed to represent all requirements of a complete EHR dataset. 59 (91%) archetypes could be found in Clinical Knowledge Manager (CKM), of which 35 could be reused directly without change, and 23 required further development including two revisions, two new versions, 18 extensions and one specialization. Meanwhile, 6 (9%) archetypes were newly developed. The legacy data of the EHR system in hospitals could be integrated into the CDR developed with these archetypes successfully.

**Conclusions:**

The existing archetypes in CKM can faithfully represent most of the EHR requirements in China except customizations for local hospital management. This case study verified the feasibility of modeling EHR with the openEHR approach and identified the fact that the challenges such as localization, tool support, and an agile publishing process still exist for a broader application of the openEHR approach.

## Background

OpenEHR is an open standard [1] maintained by the openEHR Foundation, which endeavors to convert health data from a physical form into an electronic form and ensures universal interoperability among electronic data in all forms [2]. The openEHR divides models into two levels (two-level modeling): the archetype model (AM) and the reference model (RM). It enables the semantic interoperability and data sharing of EHRs, which differentiates the representation of data instances from the domain knowledge. The openEHR approach is a multi-level single source modeling within a service-oriented software framework. It is a promising approach to facilitate the interoperation of EHR systems, which is based on the fact that a complete EHR dataset can be fully represented using sharable archetypes.

The openEHR approach has three major pillars: RM, AM, and terminology. The RM is a stable and formal information model that focuses on the logical structures of an EHR and defines the basic structures and attributes needed to express EHR data instances, including data types, data structures, and components of an EHR. The AM consists of archetypes and templates. Archetypes are the formal and semantic artifacts that facilitate collecting, storing, retrieving, representing, communicating and analyzing clinical data, which can be modeled by clinical professionals and health informatics experts by constraining RM. Meanwhile, each archetype is designed towards reuse; in other words, it should be agreed and shared to contribute to semantic interoperability among different EHR systems. An archetype should represent the maximal data set of a domain concept. The types of archetypes are listed as follows:

- Demographic: defines generic concepts of demographic information; includes PARTY, ROLE and relevant detailed classes.
- Composition: the top-level structure and “data container” containing section archetypes and entry archetypes, and it is considered equivalent as a clinical document.
- Section: a navigational structure that facilitates human access, which is similar to the table of contents of a document. A section archetype can contain section archetypes and entry archetypes.
- Entry: defines the generic structures for representing clinical statements, which has five descendants as follows:
  - Observation: represents the observations that occurred to the patient in the past, including clinical observations, examinations, lab tests and situations of the patient.
  - Instruction: represents the interventions to be performed in the future, e.g., medication orders.
  - Action: represents what has been executed, e.g., insertion of an intravenous cannula.
  - Evaluation: represents opinions and assessments on the patient, such as diagnosis, risk assessment, goals and recommendations.
  - Admin_Entry: used to capture administrative information, such as admission, appointments, discharge, billing, and insurance information.
- Cluster: represents reusable clinical content that can be embeded into entry archetypes or other cluster archetypes.
- Element: represents a single item to be reused in entry archetypes or cluster archetypes.

An openEHR template assembles and constrains archetypes for context-specific purpose, which is closest to users and typically used to generate application programming interfaces(APIs), XML schema definitions (XSDs), user interface forms, storage schemes, etc.

OpenEHR is a terminology-neutral approach, which allows referring to external terminologies in archetypes, such as SNOMED CT, ICD, LOINC and so forth. Archetype plays an important role in the openEHR approach, which not only supports representing the semantics but also facilitates maintainability [3], scalability and interoperability [4], and input from the clinical practitioners [5].

The openEHR approach adopts multi-level modeling method that clearly divides the responsibility, in other words, the technicians account for the software coding with RM, and the semantics of information is defined by the domain experts. As the openEHR approach is archetype-driven, the structure of data storage and user interface can be generated by archetypes and templates. Archetypes are computable, which means they can be generated and reused in an automated way [6]. As a result, the domain experts can participate in the development of systems through defining archetypes and binding appropriate terminology. On the other hand, due to the separation of archetypes and RM, the engineers only need to focus on developing software or systems based on the RM without considering what clinical knowledge will be involved in.

The openEHR approach has received many attentions from both industry and academy through many national or regional initiatives from many contries [7]. Recently, the achievements of the openEHR approach have been reported in many countries, such as Brazil [8, 9], Australia [10–12], Germany [13], Russia [14], Japan [15], Norway [16], UK [17, 18], Sweden [19], Denmark [20], Indonesia [21, 22], China [23] and so on. Among these countries, China is still in its infancy stage of using openEHR approach. In China, openEHR has drawn the attention of related organizations and vendors, but expectations and doubts coexist.

Archetype modeling is essential for the openEHR approach and determines the outcome of the openEHR approach implementation. To date, the feasibility of using archetypes to represent different domain contents has been described in many scenarios, such as multiple sclerosis functional composites [24], nursing [25], obstetrics [1], premature infants [13], drug management [17], biobanks [18], common data elements (CDEs) [26], regional EHR [27], quality indicators and routine patient data [28] and clinical data sets [29]. Besides, some openEHR based EHR-related implementations have been reported on the openEHR website [14], such as Shared Electronic Health record in Australia, web-based ambulatory care EHR system in Brazil, and DIPS EPR solution in Norway. However, the feasibility of archetyping a complete EHR dataset in the hospital has not reported in academic literature.

This study conducts a case study of modeling an EHR in China aiming to investigate the feasibility and challenges of archetyping a complete EHR dataset with the openEHR approach.

The research questions addressed in this study are:

- Whether the existing archetypes in the CKM can meet the requirements of a complete EHR dataset?
- What challenges will arise when modeling an EHR with the openEHR approach?

## Methods

**Fig. 1:**
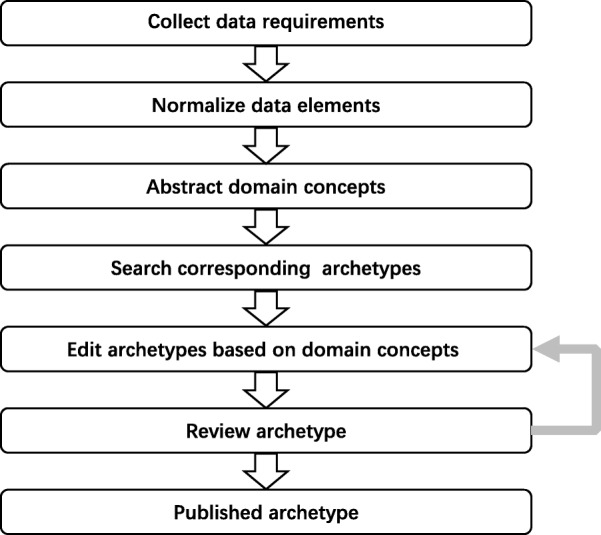
The iterative process of archetype modeling

There are several studies related to archetype modeling. Beale, Leslie, and Bakke *et al* introduced the principles and constraints of archetype modeling [30–32]; Madsen *et al* described the iterative process of archetype modeling [33] ; Buck, Spath, and Braun *et al* explained the detailed steps of converting existing information requirements into archeytpes [13, 18, 24]. To archetype a complete EHR dataset, the authors proposed an archetype modeling method referring to these studies. The method is an iterative process consists of six steps which were designed for this case study but can also be used in other cases. These steps include: collecting data requirements, normalizing data elements, organizing domain concepts, searching corresponding archetypes, editing archetypes, and reviewing archetypes (See Fig. 1).

Collecting data requirements is the first step which decides the scope and content of archetype modeling. The sources of data requirements mainly refer to existing health information systems but are not limited to them, which could also be standards, books, guidelines, journal articles or other related artifacts. Normalizing data elements refines the granularity and definition of data elements captured as the data requirements in the first step. Then, these data elements will be organized into domain concepts. Since each domain concept corresponds to one archetype, organizing domain concepts affects the quality of archetypes and should conform to domain knowledge. Searching corresponding archetypes in the archetype repository for domain concepts is essential to reuse existing archetypes as much as possible, which facilitates the archetypes sharing and semantic interoperability. According to the searching results, different rules are used in the step of editing archetypes. Finally, reviewing archetypes by domain experts is performed to acquire consensus and high-quality archetypes. These steps will be explained in more detail as follows.

### Collecting data requirements

To date, EHR systems have been adopted by most hospitals in China, especially the tertiary hospitals. The EHR systems were designed to collect, store, manage and use EHR data, which is a longitudinal electronic record of patient health information generated from one or more encounters in the care delivery institutions [34].The data within the EHR systems consists of patient demographics, progress notes, problems, medications, vital signs, past medical history, immunizations, laboratory data, radiology reports, admission discharge and transfer (ADT) and so forth. They could be taken as the reference sources for modeling a complete EHR dataset because they represent the data requirements of an EHR from actual practices.

In this study, two representative existing EHR systems were chosen as the sources to collect data requirements. One has been implemented in more than 1000 hospitals and is the most influential one in China now. The other is the system developed in the national project “R&D of High-end EHR system” (2012-2015) and has been implemented in a pilot hospital, which stands for the cutting edge EHR system in China. Both systems use relational database to store EHR data.

To collect data requirements, the relational database schemes of the two existing EHR systems have been interpreted to tables and fields. First, all the data fields in the relational tables were collected as the data elements required in an EHR. Then these data elements were grouped into the corresponding categories based on the tables they belong to.

The tables in the relational database of the EHR systems are usually designed to support specific business logics or certain functions rather than represent domain concepts. One relational table may only cover a part of attributes of one or more domain concepts and barely corresponds to one domain concept exactly. Since a requirement category was defined as a group of domain concepts with similar functions, like order information, ADT information, etc., several tables can be grouped into one category according to the function. Thus, it’s convenient to use category to group the tables first so that it contains all the involved data elements, and then organize these data elements into domain concepts further.

### Normalizing data elements

To acquire complete EHR data elements without semantic overlap, three EHR-related national standards in China were referenced, namely, “WS 363-2011 Health data element dictionary” (WS 363-2011) [35], “WS364-2011 Classification and coding for value domain of health data element” (WS364-2011) [36] and “WS 445-2014 Basic dataset of electronic medical record” (WS 445-2014) [37]. WS 363-2011 defined standardized data elements with a set of attributes, such as name, definition, data type, presentation format and allowed values. W364-2011 defined the value range of data element with coded value, meaning, and comment. WS 445- 2014 defined 17 standardized EHR data subsets commonly used in EHR domain, which can facilitate information exchanging between different systems for certain data subsets. The data elements of the standardized data subsets within WS 445-2014 were referred to the corresponding definition of the data elements in WS 363-2011, and the value range of these data elements were referred to the corresponding definition of coded value in W364- 2011.

As these three standards are defined to facilitate the data interoperability by collecting and analyzing existing clinical business forms from some representative hospitals in China, they only cover part of the EHR data requirements for interoperability rather than all the EHR data requirements. As a result, mismatches might exist between the standards and EHR data requirements from actual clinical practices. Despite the mismatches exist between these data standards and EHR data requirements, these data standards can still help normalize the data elements, including data elements complement and normalization.

First, a complete set of data elements was acquired by complementing the data elements that were not included in the data requirement categories in the previous step but were defined within these standards.

Then, the data elements of EHR requirements were normalized by referencing these three standards. The rules for the normalization process are listed as follows:

- If an EHR data element and a standard data element have the same semantics, then the EHR data element uses the definition of the standard data element as the normalized definition, including naming, value domain, coded value, and comments.
- If multiple EHR data elements correspond to one standard data element, which means that the granularity of EHR data elements is finer than those of the standards, then these EHR data elements and the corresponding standard data element will both be reserved.(e.g., the Apgar score corresponds one standard data element, while it corresponds to six EHR data elements)
- If an EHR data element corresponds to multiple standard data elements and the semantics of the EHR data element can be represented entirely by these standard data elements, then the EHR data element is replaced by the standard data elements. (e.g., the address data is recorded with one narrative data element of EHR, while it is consisted by six standard data elements, including province, city, county, street and door number.)
- If one EHR data element corresponds to several standard data elements and the semantics of the EHR data element cannot be represented entirely by these standard data elements, then the EHR data element and these standard data elements will all be reserved.
- If multiple EHR data elements correspond to mutliple standard data elements with semantic overlap among them, then a discussion will be carried out based on the premise of reserving these standard data elements.

### Organizing domain concepts

Based on the categories of EHR requirements and the normalized data elements described in previous steps, domain concepts were organized with three patterns: patient demographics pattern, clinical pattern, and non-clinical pattern. Each EHR requirement category corresponds to one of these three patterns.

For the patient demographic pattern, the experience of EHR system implementation was used to organize the concepts. The concepts include patient information, address, and organization.

For the non-clinical pattern, the process of encounter was considered to organize the concepts. Following concepts can be organized: admission, discharge, and transfer.

For the clinical pattern, the problem-solving logic that represents the cycle of clinical information flow is used to organize the concepts. The problem-solving logic divided clinical information into four types of “Instruction, Action, Evaluation, and Observation”. The “Instruction” type corresponds to the information about intervention plan, which will happen in future. The “Action” type represents what has happened about the intervention. The “Observation” type is all about the objective observation data, such as laboratory test result, ECG report, and imaging examination result. The “Evaluation” type is about opinion and summary, which is always given by care providers, such as diagnosis information, health risk assessment, and social summary. Every coarse EHR requirement category with clinical pattern was divided into finer clinical concepts based on the problem-solving process. For example, the “imaging examination” category was divided into concepts of imaging examination request, imaging examination action, imaging examination result and imaging series. Clinical experts were invited to review the organized concepts in this step. To help clinical experts to judge the feasibility and rationality of these clinical concepts effectively, these domain concepts were illustrated as mind-maps.

### Searching corresponding archetypes

To reuse existing archetypes as much as possible, the searching step was executed to find out the corresponding archetypes for the domain concepts, which is of great significance for semantic interoperability. In addition, the searching step can also facilitate the improvement of domain concepts by referencing existing archetypes.

The corresponding existing archetypes were retrieved based on three kinds of relationships between existing archetypes and domain concepts. First, the concept and the existing archetype have the same semantics, e.g., the concept “diagnosis” and the existing archetype “openEHR-EHR-EVALUATION.problem_d-iagnosis.v1”. Second, the semantics of the concept is one of the particular subsets of the existing archetype semantics, e.g., the concept “operation request” and the existing archetype “openEHR-EHR-INSTRUCTI-ON.request.v0”. Third, the semantics of the concept is more general than the existing archetype’s, e.g., the concept “physical sign” and the existing archetype “openEHR-EHR-OBSERVATION.body_temperature.v2”.

As for the third one, the domain concepts were refined, e.g., five new concepts was refined to detail physical sign information, including height, weight, body surface area, body mass index, and body temperature.

Based on these three kinds of relationships, the CKM was adopted as the source to search corresponding existing archetypes for domain concepts. The CKM, supported by the openEHR Foundation, is a repository for incorporating development, management, publishing and sharing a wealth of clinical knowledge with the international openEHR community. Since only nine archetypes in CKM have Chinese version, it is necessary to translate the domain concepts into English before searching. Given that string matching is the cornerstone of the CKM search function, the accuracy of translation affected the corresponding search result directly. To improve the accuracy and recall ratio of the search operation, the synonyms were considered as much as possible. To facilitate the reuse of existing archetypes, manual searching was performed. Although the manual search operation could promote existing archetype reusability by improving the accuracy of the archetype search result, it was time-consuming and laborious.

For each domain concept, the CKM based searching was executed with the domain concept name, data items, and synonyms respectively. Then, the existing archetypes related to the domain concept were identified by comparing the content of the concept and archetypes, involving the meta-data, the definition, and ontology. When the search results contain one or more of the existing archetypes, the one with the highest similarity of matching was chosen for reuse. After that, the final version domain concepts and corresponding existing archetypes were confirmed.

### Editing archetypes based on domain concepts

The domain concepts and corresponding existing archetypes were compared, and further divided the results into six categories. Then the rules to edit archetypes were designed according to these six categories, as shown in Table 1.

**Table 1:** The mapping rules for archetype editing

Result of searching	Category of covered	Operation
	Covered by archetype completely.	Used directly
	Need to modify description, translation, and extend the value sets.	Revision
Existing corresponding archetype	Need to specialize the archetype and add more constraint.	Specialization
	Need to add new items to the definition section and maintain compatibility.	Extension
	Modification that makes the archetype incompatible with the original archetype.	New version
No corresponding archetype	No covered	New

The domain experts would define new archetypes according to clinical concepts if there were no corresponding archetypes in CKM. Correspondingly, domain experts would execute five kinds of operations for archetype reuse when the clinical concept has matched archetypes in CKM:

- If the existing archetype covered all the data elements and nothing needed to be changed, then the existing archetype was reused directly.
- If the existing archetype covered all data elements but the meta-data required to be refined, then a revision operation was executed, including translation, extending value sets and description.
- If the existing archetype covered only a portion of the data elements, three potential modification choices were provided. The specialization operation was executed when the clinical concept could be expressed by specializing the existing archetypes to make the semantics more elaborate and narrow, which required changing the identification information of the archetype. The extension operation was executed when some compatible modifications to the existing archetype were needed for expressing the clinical concept. A new version was created when some incompatible modifications to the existing archetype were needed, which changed the version information.

The modification of existing archetypes referred to modifying meta-data, adding data elements, and adjusting the value range as well as terminologies. When a new archetype requires to be designed, a suitable archetype type and a proper archetype name should be chosen first. Then, the meta-data of the archetype was edited, including concept description, keywords, purpose, use, and misuse. At last, the data element and relevant terminologies were edited.

There are some editting tools capable of facilitating archetype modelling, such as Archetype Editor (AE) [38], LinkEHR Editor [39] and LiU Archetype Editor [40]. AE is more readily accepted by users, with a graphic user interface and drag-and-drop editing mode, but it does not support demographic archetypes editing. LinkEHR Editor allows editing all information about archetype, but the user interfaces are more engineer-oriented than AE, which may confuse users who lack technical knowledge. Given these facts, this study used the AE and LinkEHR Editor in different scenarios. LinkEHR Editor accounted for editing demographic archetypes, while AE took charge of editing others.

### Reviewing archetypes

Reviewing archetypes is a pragmatic way to acquire consensuses and high-quality archetypes within the target domain, which is always executed by domain experts. In this method, two kinds of archetype statuses were designed, i.e. “initial” and “published”. The archetype with initial status is an initial or intermediate artifact, but the published archetype is the final product that can be implemented within EHRs. An archetype’s status can only change to published when it passes the review step. Otherwise, the archetype will go back to the previous step, and the iterative process will be executed until domain experts successfully authorize the archetype.

To facilitate the quality improvement of archetype modeling and make archetypes sharable and reusable, a review group was organized to perform the review process. The group reviewed two aspects of the defined archetypes: domain concepts and information representation. For the domain concepts review, the archetypes were represented as mind-maps initially for facilitating domain experts review. Then, the meta-data and organizational structure of the concept was evaluated, including naming, description and terminology constraints, and the relationships between the data items. For the information aspect, the review focused on the choice of data type and the organization of data items.

## Results

The case study is started from analyzing two existing EHR systems. The data elements from these two systems have been collected and further grouped into 13 corresponding categories. The categories and the number of data elements within are illustrated in Table 2.

**Table 2:** Details of EHR data requirement collection

Database scheme-1	Database scheme-2	EHR requirements
PAT_MASTER_INDEX	MASTER_PATIENT_INDEX	Patient demographics (69 items)
MEDREC.DIAGNOSIS	DIAGNOSIS	Diagnosis information (25 items)
MEDREC.PAT_VISIT	PATIENT_VISIT	
OUTPADM.CLINIC_MASTER	VISIT_IN_HOSPITAL	ADT information (175 items)
INPADM.PATS_IN_HOSPITAL	VISIT_OUT_PATIENT	
ORDADM.ORDERS	ORDERS	Order information (92 items)
OUTPDOCT.OUTP_ORDERS	ORDERS_PERFORM	
ORDAMD.VITAL_SIGNS_REC	VITAL_SIGNS_RECORD	Vital signs (17 items)
EXAM.EXAM_MASTER	EXAM_REQUEST	
EXAM.EXAM_ITEMS	EXAM_ITEM	Imaging examination (182 items)
EXAM.EXAM_DATA	EXAM_DATA	
EXAM.EXAM_REPORT	EXAM_REPORT	
LAB.LAB_TEST_MASTER	LAB_TEST_REQUEST	
LAB.LAB_TEST_ITEMS	LAB_TEST_DATA	Lab test (112 items)
LAB.LAB_RESULT	LAB_TEST_MASTER	
OPERATION_SCHEDULE	OPERATION_REQUEST	Operation information (200 items)
OPERATION_MASTER	OPERATION_REPORT	
BLDBANK.BLOOD_APPLY	None	Transfusion (36 items)
BLDBANK.BLOOD_CAPACITY		
NURSERCORD_SUMMARY	None	Nursing information (62 items)
None	CONSULT_MASTER	Consult information (19 items)
None	NEWBORN_REPORT	Newborn information (129 items)
EMR.EMR_DOCUMENT	EMR_DOCUMENT	EMR document information (88 items)
	EMR_DOCUMENT_DETAIL	
Total		1226 items

Then the normalization based on the existing national standards was performed. After a detailed analysis and organization, 91 data elements have been complemented to the 13 categories to acquire a complete EHR dataset, which are illustrated in Table 3. After that, totally 932 data elements have been normalized to represent the data requirements of a complete EHR dataset in China. Details of the structured data items are illustrated in Table 3.

**Table 3:** The results of data elements normalization

EHR requirements	Data scheme-1	Data scheme-2	EHR data elements	Complemented data elements from standards
Patient demographics	29	44	48	7
Diagnosis information	12	13	15	2
ADT information	109	66	123	11
Order information	43	49	105	34
Vital signs	7	10	12	3
Imaging examination	103	79	113	5
Lab test information	48	64	66	3
Operation information	83	117	124	3
Transfusion	36	None	42	6
Nursing information	62	None	66	4
Consult information	None	19	23	4
Newborn information	None	129	132	3
EMR document	33	55	63	6

**Fig. 2:**
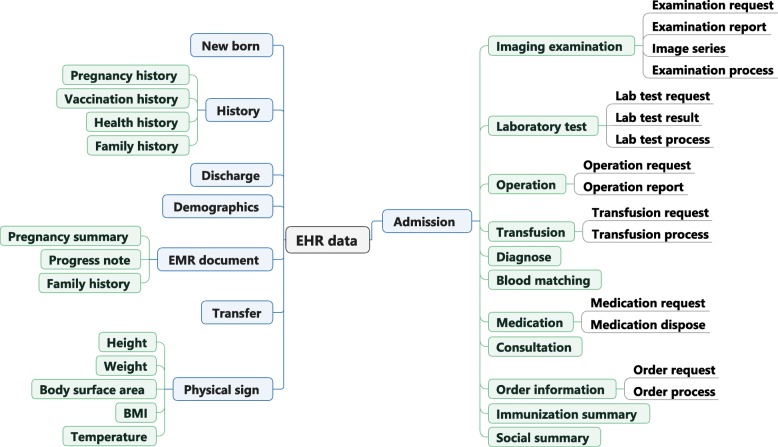
The results of concepts abstraction. We classified 37 clinical concepts guided by the reference model of openEHR and based on the clinical practices in China

Those data elements have been organized into 37 concepts in the third step, which are illustrated in Fig. 2.

In the fourth step, we searched the existing archetypes in CKM for the domain concepts. The searched results are illustrated in Table 4.

**Table 4:** The searched results of domain concepts

Domain concept	Corresponding existing archetypes
Dmographics	openEHR-DEMOGRAPHIC-ADDRESS.address.v1
	openEHR-DEMOGRAPHIC-ADDRESS.electronic_communication.v1
	openEHR-DEMOGRAPHIC-PERSON.person.v1
	openEHR-DEMOGRAPHIC-PARTY_IDENTITY.person_name.v1
	openEHR-DEMOGRAPHIC-ORGANISATION.organisation.v1
	openEHR-DEMOGRAPHIC-CLUSTER.person_identifier-provider.v1
	openEHR-DEMOGRAPHIC-CLUSTER.person_identifier.v1
	openEHR-DEMOGRAPHIC-PERSON.person-patient.v1
	openEHR-DEMOGRAPHIC-ITEM_TREE.person_details.v1
Immunization summary	openEHR-EHR-EVALUATION.Immunization_summary.v1
Social summary	openEHR-EHR-EVALUATION.social_summary.v1
Pregnancy history document	openEHR-EHR-COMPOSITION.pregnancy_summary.v1
Pregnancy history	openEHR-EHR-EVALUATION.pregnancy_summary.v0
	openEHR-EHR-CLUSTER.document_entry_metadata.v1
Vaccination history	openEHR-EHR-EVALUATION.vaccination_summary.v1
Health history	openEHR-EHR-EVALUATION.health_risk.v1
Family history document	openEHR-EHR-COMPOSITION.family_history.v1
Family history	openEHR-EHR-EVALUATION.family_history.v2
	openEHR-EHR-EVALUATION.exclusion-family_history.v1
	openEHR-EHR-CLUSTER.person_name.v1
Progress note document	openEHR-EHR-COMPOSITION.progress_note.v1
Progress note	openEHR-EHR-EVALUATION.absence.v1
	openEHR-EHR-CLUSTER.distribution.v1
	openEHR-EHR-EVALUATION.exclusion.v1
	openEHR-EHR-OBSERVATION.progress_note.v1
Admission	openEHR-EHR-ADMIN_ENTRY.admission.v1
	openEHR-EHR-CLUSTER.address.v1
	openEHR-EHR-CLUSTER.organisation.v0
	openEHR-EHR-CLUSTER.education.v1
	openEHR-EHR-CLUSTER.household.v0
	openEHR-EHR-CLUSTER.employment.v0
Discharge	openEHR-EHR-ADMIN_ENTRY.discharge_admin_info.v3
Blood matching	openEHR-EHR-OBSERVATION.blood_match.v1
Transfusion request	openEHR-EHR-INSTRUCTION.transfusion.v0
Transfusion process	openEHR-EHR-ACTION.transfusion.v1
Medication request	openEHR-EHR-INSTRUCTION.medication_order.v1
	openEHR-EHR-CLUSTER.medication_ingredients.v1
	openEHR-EHR-CLUSTER.medication_admin.v1
	openEHR-EHR-CLUSTER.timing.v1
	openEHR-EHR-ACTION.medication.v0
Lab test request	openEHR-EHR-INSTRUCTION.request-lab_test.v1
	openEHR-EHR-CLUSTER.specimen.v0
Lab test process	openEHR-EHR-INSTRUCTION.lab_test.v1
Lab test result	openEHR-EHR-OBSERVATION.lab_test.v1
Newborn	openEHR-EHR-OBSERVATION.apgar.v1
Height	openEHR-EHR-OBSERVATION.height.v1
Weight	openEHR-EHR-OBSERVATION.body_weight.v2
Body surface area	openEHR-EHR-OBSERVATION.body_surface_area.v0
BMI	openEHR-EHR-OBSERVATION.body_mass_index.v2
Temperature	openEHR-EHR-OBSERVATION.body_temperature.v2
Consultation	openEHR-EHR-INSTRUCTION.request.v0
Order request	openEHR-EHR-INSTRUCTION.request.v0
Order process	openEHR-EHR-ACTION.procedure.v1
Operation request	openEHR-EHR-INSTRUCTION.request-procedure.v0
Operation report	openEHR-EHR-OBSERVATION.operation_record.v1
Examination request	openEHR-EHR-INSTRUCTION.request-imaging_exam.v1
Examination report	openEHR-EHR-OBSERVATION.imaging_exam.v0
Examination process	openEHR-EHR-ACTION.imaging_exam.v0
Diagnose	openEHR-EHR-EVALUATION.problem_diagnosis.v1

After that, the new archetypes and the need to be modified archetypes were edited using the tools of AE and LinkEHR Editor. Finally, these archetypes were reviewed by the review group that comprises two medicine professors who have more than 10 years clinical service experience, two medical informatics professors who take part in biomedical research more than 10 years, one medical informatics expert who works in medical informatization construction for more than 10 years, one clinical data integration expert who has six years clinical data integration experience in actual clinical practice. Every domain expert gave their review comments for each archetype, and these comments were collected as the materials for discussion in the archetype review seminar. Four seminars were held to reach a consensus on all the review results, and each seminar lasted two days.

**Fig. 3:**
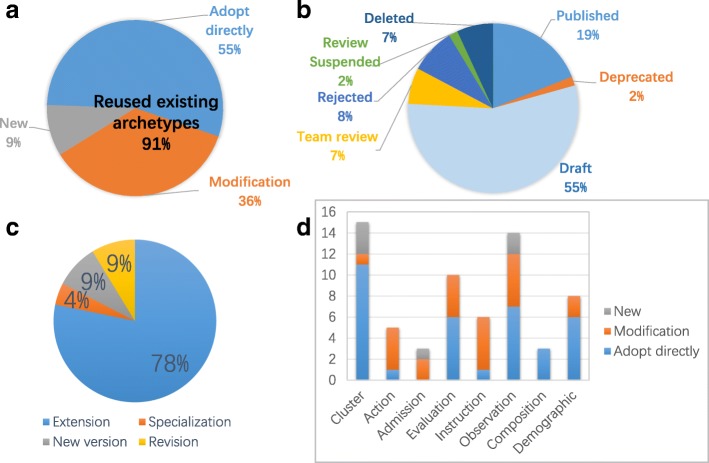
The data statistics of the archetypes developed in this study. **a** The distribution of new, direct adoptions and modifications across all the developed archetypes in this study. **b** The status information distribution of all reused archetypes. **c** A pie chart of the existing archetype modifications. **d** A histogram of all developed archetypes, each of which represents an archetype type. The length of each bar indicates the number of archetypes from a specified source, including new, modification and adopt directly

After one-year endeavor, 64 archetypes were developed (see Table 5) to cover the requirements of an EHR. Across all the archetypes, 55% (35) were adopted directly from CKM, 9% (6) were new created, and 36% (23) were modified based on the existing archetypes. In other words, 91% of archetypes came from reusing existing archetypes (see Fig. 3a). By analyzing the status data of the reused archetypes (see Fig. 3b), we found that published archetypes only accounted for 19%, which means that most of the reused archetypes had not been approved. Meanwhile, the Rejected, Deprecated and Deleted archetypes accounted for 17% of the reused archetypes, which means that these archetypes were not advised to use. The modified archetypes consisted of two revisions, two new versions, one specialization and 18 extensions. There were 78% of modified archetypes developed through extending existing archetypes (see Fig. 3c). In addition, we found that modifications occurred mostly in the action, admission, evaluation, instruction and observation archetypes; direct adoptions appeared mostly in the cluster, evaluation, observation and demographic type archetypes; new archetypes appeared in the cluster, admission, and observation type archetypes (see Fig. 3d).

**Table 5:** Archetypes for a complete EHR dataset in China

Adopt directly	Modification	New
CLUSTER.timing	DEMOGRAPHIC-PERSON.person-patient	CLUSTER.insurance
CLUSTER.medication_admin	DEMOGRAPHIC-ITEM_TREE.person_details	CLUSTER.electronic_communication
CLUSTER.medication_ingredients	ACTION.imaging_exam	CLUSTER.contacts
CLUSTER.address	ACTION.Lab_test	ADMIN_ENTRY.transaction
CLUSTER.distribution	ACTION.medication	OBSERVATION.physical_sign
ADDRESS.electronic_communication	ACTION.transfusion	OBSERVATION.imaging_exam_image_series
CLUSTER.organization	ADMIN_ENTRY.admission	
CLUSTER.specimen	ADMIN_ENTRY.discharge_admin_info	
DEMOGRAPHIC-PERSON.person	EVALUATION.problem_diagnosis	
DEMOGRAPHIC-PARTY_IDENTITY.person_name	INSTRUCTION.medication_order	
DEMOGRAPHIC-ORGANISATION.organization	INSTRUCTION.request-imaging_exam	
DEMOGRAPHIC-CLUSTER.provider_identifier	INSTRUCTION.request-lab_test	
DEMOGRAPHIC-CLUSTER.person_identifier	INSTRUCTION.request-operation	
INSTRUCTION.request	INSTRUCTION.transfusion	
OBSERVATION.lab_test	OBSERVATION.operation_record	
CLUSTER.document_entry_metadata	OBSERVATION.imaging_exam_report	
COMPOSITION.pregnancy_summary	OBSERVATION.lab_test_single	
COMPOSITION.progress_note	EVALUATION.pregnancy_summary	
CLUSTER.person_name	EVALUATION.vaccination_summary	
COMPOSITION.family_history	OBSERVATION.progress_note	
EVALUATION.family_history	OBSERVATION.blood_match	
EVALUATION.exclusion-family_history	EVALUATION.immunisation_summary	
EVALUATION.absence	CLUSTER.employment	
EVALUATION.health_risk		
EVALUATION.social_summary		
CLUSTER.education		
CLUSTER.household		
OBSERVATION.body_surface_area		
OBSERVATION.body_mass_index		
OBSERVATION.body_weight		
OBSERVATION.height		
ACTION.procedure		
EVALUATION.exclusion		
OBSERVATION.body_temperature		
OBSERVATION.apgar		

The newly developed archetypes were used to represent the domain concepts that were not covered by existing archetypes in CKM, which included three cluster type archetypes, one admin_entry type archetype and two observation type archetypes. The cluster archetypes were designed to represent the information reused within other archetypes, including insurance information, electronic communication information, and contact information. Although the electronic communication and contact information were defined in the demographics, these archetypes could not work in the EHR type archetypes. Also, we developed the insurance cluster archetype to represent the insurance information. Transfer information is a necessary part of administration information in EHRs in China that is not represented by the archetypes in CKM. An admin_entry archetype was designed to record the transfer information between different departments or hospitals according to the transfer requirements from the two existing EHR systems. These two observation type archetypes were developed to represent the physical sign information and image series information. Although some existing archetypes in CKM could describe physical sign data, they were designed to represent specific physical sigh, such as blood pressure, temperature, and heartbeat. While the physical sign information was not limited to these archetypes in CKM, and it can be different and specialized in diverse hospitals in China. Therefore, to represent the complete physical information, we developed a new archetype to express the general concept of physical signs. The relationship between the imaging examination report and imaging study might be one-to-many, but the existing imaging examination archetype within CKM describes the relationship between the imaging report and imaging study as one-to-one. Considering the relationship representation and the definition of image information according to DICOM standard, we developed a new observation archetype to represent the imaging study, imaging series and the one-to-many relationship between the imaging report and imaging study. The requirement of relationship representation between imaging report and imaging study has been submitted to the international CKM through the Change Request features. In addition, the author has fed back the idea of developing a new archetype for image information to international CKM via the Discussion features of CKM, which can help identify whether it is an undiscovered international requirement or a localized requirement of Chinese clinical practice. These feedbacks to the international community CKM are both taken advantage of the well-established open source/distributed development processes of CKM.

Three confusions about information representation have been solved in the modeling process, which involved participation information, relationship information and identification information.

First, although the specifications of openEHR illustrated that there is an “otherParticipations” attribute in RM can represent participation information, all the corresponding archetypes found in CKM recorded participation information with the protocol attribute rather than the “otherParticipations” attribute. Then we chose to follow the existing archetypes’ style.

Second, with regard to relationship representation, there is no clear pattern on how to express the relationship between archetypes, specifically, representing relationships between entry classes, including admin entry, instruction, evaluation, observation, and action. Although openEHR provides a slot and link mechanism to express relationships, they do not work well. On the one hand, the slot mechanism cannot work between entry class archetypes. On the other hand, the link mechanism lacks details about how to use it. After several discussions, we use the link mechanism to represent the relationships between entry-level archetypes by adding links into a target archetype with the identification and the path of the source archetype. For example, we edited a link into openEHR-EHR- INSTRUCTION.request-imaging exam.v1 to represent the relationship that one admission may correspond to many imaging examinations by referencing the encounter identifier information of openEHR- EHR-ADMIN ENTRY.admission.v1. In addition, the team determined that using standardized terminology in an archetype is challenging because Chinese terminology standardization lags far behind the development of health information technology in China. As hospitals did not adopt unified international terminology, in this case, the modeling team used the private terminology of the hospital, which is similar to the EHR-AECHE project [41].

Third, although the subject identifier information can be recorded even if the subject identifier element is not defined in the entry archetype, the subject identifier element was defined explicitly in each entry archetype in consideration of archetype review and the GUI generation. Theoretically, ENTRY class and its subtypes all have a subject attribute for recording the subject of the Entry record, and entry archetypes are defined by constraining them, so there is no need to define the subject identifier within each entry archetype just for recording the subject information in a working system. However, in this case study, the integrity review of the concept given by clinical experts and the semi-automatically generation of GUI involving subject identifier configuration require the subject identifier to be expressed explicitly within entry archetypes.

During the modeling process, we found that some reused archetypes had been deprecated in CKM. For example, the “openEHR-EHR-CLUSTER.-medication_-admin.v1” and “openEHR-EHR-CLUSTER.-medicatio-n_amount.v1” were in the draft status when the modeling team adopted them, but now they have been deprecated by CKM. Also, the “openEHR-EHR-ADMIN_ENTRY.discharge_admin_info.v3” archetype was in the draft status when the modeling team reused it, but it is now missing from CKM.

To verify the feasibility of the openEHR approach and the usability of these archetypes for an EHR, we implemented 64 archetypes in a CDR in a tertiary hospital. A database scheme consists of 80 relational database tables were generated with the archetype relational mapping method [23], and 164 APIs were generated from these archetypes. Using these APIs, we integrated the legacy data of these two existing EHR systems into the CDR and ran two clinical data applications on these data, which illustrated that the archetypes developed in this study can represent the EHR data requirements effectively.

## Discussion

The CKM and archetype modeling method facilitates the openEHR approach. On the one hand, CKM has accumulated enough archetypes to cover most of the EHR requirements, which supports archetype sharing and reuse. On the other hand, the archetype modeling method enables the domain experts to design archetypes to represent their requirements, especially, new requirements not covered by existing archetypes. To meet the EHR requirements in China, some existing archetypes were reused through different modification operations, and some new archetypes were developed from scratch. Based on the localized EHR requirements, the modifications consisted of language translation, value range adjustment, data elements supplementation, data elements specialization and so on. Also, we explicitly defined subject identifier and link attributes to represent patient identification and relationships in archetypes. Then, the newly developed archetypes were comprised of four categories corresponding to localization requirements, including reusable cluster archetype definitions, administration information supplementation, granularity adjustment, and relationship representation. As these modifications and new archetypes are closely aligned to a mix of legacy EHR data tables and a set of national standards in China, it is possible that some of new archetypes and modifications are generally re-usable in China rather than worldwide.

These EHR archetypes have been uploaded to the Healthcare Modeling Collaboration (HMC) [42] that has a governance model to facilitate other hospitals to reuse the same archetypes in China. These archetypes are used by Shanxi Dayi hospital to implement a clinical data repository, which is used to store and manage the EHR data for access and retrieval by other information systems or applications. Besides, there is one vendor using these archetypes to develop a regional healthcare platform that aims to share and utilize the EHR data. As these archetypes cover the EHR requirements rather than common interoperability requirements, they can be an important reference resource to facilitate the development of national standards.

Through this case study, we learned some lessons from the openEHR archetype modeling of an EHR in China.

### The lessons related to archetype modeling

Although some modifications required to be made, the existing archetypes in CKM covered most of the EHR requirements. These modifications may reflect two type of requirements: the localization requirements in China and the international requirements that have not come across. The timely feedback to the international CKM is one of the effective means of identifying the type of requirements. The localization requirements can help to promote the development of openEHR localization. If the feedbacks are the international requirements, they can facilitate the development and reuse of international archetypes. Most of these modifications were archetype extensions; only a few were revisions, new versions, and specializations. These modifications appeared mostly in the entry archetypes, including action, admission, evaluation, instruction, and observation. These modifcations In contrast, the direct adoption of existing archetypes appeared in the cluster, demographic, evaluation and observation archetypes. Authors think that there are three reasons for this. First, most of the reused evaluation and observation archetypes are in the published state. Second, the demographic requirements are similar in different clinical practices. The last but not the least, the granularity of cluster is enough fine to reuse.

Granularity selection is a challenge to archetype modeling. On the one hand, the fine-grained archetype represents information with precise semantics, but the information that it can express is relatively narrow. In contrast, the coarse-grained archetype can represent a broader range of information, but it will lose some semantics compared with the fine-grained one. The localization of openEHR archetypes should be balanced between the completeness of the information and the granularity of the semantics. In this study, we developed a new observation archetype to represent the general physical sign concept rather than developing more fine-grained archetypes to express the specialized physical sign concepts. The reason why we did this is that the physical sign information requirements from the two EHR systems could be hardly divided into distinct specialized archetypes.

The relationship between concepts may not be the same among different countries, as the actual clinical practices are different. In this study, we found that the relationship between the imaging report and imaging study was one-to-many rather than one-to-one, as represented in the imaging examination archetype within CKM. To express this relation, we developed a new archetype to represent the imaging study information and then used the link function to build this one-to-many relationship. It is better to represent the one-to-many relationship by splitting one archetype into two standalone archetypes and building a relationship between them. In addition, these new requirements were fed back to CKM, which might be useful to international archetype development.

Although the translation is time-consuming and laborious, it is a necessary and essential task that affects archetypes definition quality and the implementation. The translation should be done before searching existing archetypes and reviewing archetype steps. The accuracy of translation influences the reusability and quality of archetypes. When searching archetypes, if the translation is not correct or appropriate, the search operation will have lower recall and precision. As a result, some corresponding archetypes will be omitted, and it will hinder the archetype reuse. During the review process, translation problems can confuse domain experts, which will jeopardize the review. Furthermore, the translation from a foreign language to mother tongue will facilitate the implementation of the archetype without understanding problems caused by language issues. However, taking into consideration the gap between information technology (IT) knowledge and clinical knowledge, accurate translation of domain concepts or archetypes is a noticeable challenge. Given the cultural and clinical practice differences and the linguistic issues, the participation of the original authors of archetypes and CKM core team might facilitate to overcome this challenge.

### The issues related to CKM and Modeling support tools

Rigorous definitions and governance are needed to facilitate archetype sharing and reuse for semantic interoperability [33]. In this concern, archetype management platforms came into being, such as openEHR CKM [43] and NHS-CfH repository [44]. The openEHR CKM, as an international archetype repository for archetype management and reuse, has been used widely [1, 23, 24, 26, 45–48] around the world. Although the openEHR CKM has accumulated nearly 500 archetypes for the most common international requirements, some modification and newly development require to be developed to meet the localized requirements of different countries. Besides CKM, there are several CKM instances for localization, including Australian CKM [49], Apperta CKM [50], Norwegian CKM [51], Slovenian CKM [52], Alberta CKM [53]. The openEHR CKM aims to represent the common part of the international EHR requirement; then, the localized CKM instance aims to satisfy the EHR requirements in their own countries. It is necessary to build a Chinese CKM to facilitate the clinical modeling in China, and the Chinese CKM should establish cooperation with the international CKM.

The slow publishing process has become an issue limiting semantic interoperability enabled by the openEHR approach. Although CKM has many archetypes to represent most of the EHR requirements, and some of these archetypes have been implemented in projects and programs, only a small portion of archetypes are at the published status. And the speed of publishing archetypes is slow and far behind the implementation. Implementing the archetypes that are not in published state may hinder the semantic interoperability, because the semantic of these archetypes may change caused by replacing, deleting or deprecating. It is necessary to accelerate the archetype publishing process to facilitate archetype sharing and semantic interoperability. In consideration of the CKM publication process is community-driven, the instant feedback of requirements from community members to CKM can speed up the process of archetype publication. Furthermore, organizing a stable domain experts group to participate in the archetype publication process may also accelerate archetype publication.

Archetype tools play an important role in the archetype editing process, which can help users define and view archetypes with a graphical user interface. AE and LinkEHR Editor are two mainstream archetype tools that are recommended by the openEHR Foundation and can be download from the openEHR official site. Although both tools can define archetypes and have been used by many projects as well as research studies, it is necessary to improve them to facilitate domain experts to participate in archetype modeling. The AE supports a drag-and-drop editing function that helps users to edit archetypes in a What You See Is What You Get (WYSIWYG) manner; however, it does not support the definition of demographic archetypes. To improve the feasibility of AE tools, at a minimum, the demographic archetypes definition function requires to be added. The LinkEHR Editor supports several kinds of RM and corresponding archetype editing [39] and allows users to edit any attributes of RM. On the one hand, users can add more constraints on RM than AE, in other words, LinkEHR Editor is more flexible than AE. On the other hand, the excessively flexible mechanism and right-click editing pattern makes clinical experts feel confused as well as requires much more IT knowledge. In conclusion, these two archetype tools both have their strengths and weaknesses, and synthesizing their strengths will help clinical experts edit archetypes effectively and efficiently.

The search function is significant for archetype reuse and affects the degree of reuse. The search function of CKM is based on string-match rather than semantic-match, which means that some corresponding archetypes may be omitted. As the current search function does not support semantic retrieval, some existing archetypes that matched the domain concept were not retrieved and reused. Therefore, to facilitate the sharing and reuse of existing archetypes, semantic-based search functions should be encouraged.

## Conclusions

By conducting a case study of modeling an EHR with the openEHR approach in China, the feasibility of modeling an EHR with the openEHR approach was verified. In this study, we found that the existing archetypes in CKM can cover most of the EHR requirements, and only a small number of archetypes were developed for localization. The newly developed archetypes corresponded to several local concepts from actual practice, such as insurance, transfer, physical signs and imaging series. Also, we found that some challenges exist for a broader application of openEHR archetyping: the domain knowledge input should be as much as possible; the publishing process of archetypes should be faster; modeling tools should be easy-to-use; the search function and the translation should be more accurate. These challenges are not unique to the openEHR approach, but they are the common problems confronted by all the attempts to develop directly implementable semantic artifacts in an open-source, distributed development manner in healthcare. This study gives some lessons and experiences to the research about archetype modeling and openEHR approach.

## Abbreviations

ADT: Admission discharge and transfer
AE: Archetype editor
AM: Archetype model
CDEs: Common data elements
CDR: Clinical data repository
CKM: Clinical knowledge manager
EHR: Electronic health record
HMC: Healthcare modeling collaboration
IT: Information technology
RM: Reference model
WYSIWYG: What you see is what you get
XSCs: XML schema definitions
